# Vaccination Is Associated With Shorter Time to Target Cycle Threshold Value in Patients With SARS-CoV-2 Omicron Variant

**DOI:** 10.3389/fcimb.2022.943407

**Published:** 2022-07-06

**Authors:** Jiajun Wu, Yong Wei, Feng Shen, Shun Zhu, Yingying Lu, Xue Tian, Pengyu Zhang

**Affiliations:** Department of COVID-19 for Temporary Centralized Isolation and Treatment, Shanghai General Hospital, Shanghai Jiao Tong University School of Medicine, Shanghai, China

**Keywords:** omicron variant, SARS-COV-2, vaccination, cycle threshold, inactivated vaccines

## Abstract

**Background:**

Limited data are available on the responses to vaccination for severe acute respiratory syndrome coronavirus 2 (SARS-CoV-2) Omicron variant in the Chinese population. This study aimed to investigate whether vaccination could alter the disease course of SARS-CoV-2 Omicron variant.

**Methods:**

A retrospective cohort included 142 patients who had no or mild symptoms and were admitted to our department for centralized isolation after being locally infected with SARS-CoV-2 Omicron variant from March 4 to 30, 2022, in Shanghai, China.

**Results:**

Of the 142 subjects with the mean age of 43.1 years, 53.5% were male and 90.8% had been vaccinated before the infection. Comparing the vaccinated with the unvaccinated patients, there was no difference in patient characteristics, but patients with vaccination had shorter time to target cycle threshold value (TtCT) (vaccinated vs. unvaccinated, 12.6 ± 3.4 vs. 14.8 ± 4.7 days, P = 0.039). There was no difference in TtCT between heterogeneous and homologous vaccination. Of subjects with homologous vaccination, 43.1% were vaccinated with CoronaVac (Sinovac Life Science), 47.2% with Sinopharm BBIBP-CorV, 4.9% with Sinopharm WIBP, 3.3% with CanSinoBio, and 1.6% with Zhifei Longcom. No difference in TtCT was observed among different vaccines. Comparing two-dose primary vaccination with three-dose booster vaccination, we found no difference in TtCT either.

**Conclusion:**

Vaccination is associated with shorter TtCT in patients with SARS-CoV-2 Omicron variant.

## Introduction

The novel severe acute respiratory syndrome coronavirus 2 (SARS-CoV-2) has spread worldwide for more than 2 years. It threatens our health and lives and seriously disrupts the daily life. The SARS-CoV-2 keeps mutating, resulting in a number of variants (Cui et al., 2019; Yang et al., 2022). The Omicron variant BA.2 is the newest variant and has become dominant in the SARS-CoV-2 outbreak in Shanghai, China, on March 2022. SARS-CoV-2 Omicron BA.2 shows an increase in its transmissibility and becomes less virulent than the Delta SARS-CoV-2 variant, apparently with less involvement of the lower respiratory tract, milder symptoms, and fewer probability of hospitalizations (Menni et al., 2022; Davies et al., 2022).

Although the SARS-CoV-2 vaccines currently have been reported to reduce the incidence of severe cases after infection (Tanriover et al., 2021), it is unclear whether vaccination could alter the natural history of SARS-CoV-2 Omicron variant infection, especially for SARS-CoV-2 vaccines made in China. Nucleic acid detection has been the primary laboratory diagnostic method for SARS-CoV-2. Both N and ORF genes of SARS-CoV-2 show significant curve, which is specific for SARS-CoV-2–positive patients. They are of great clinical significance for the cycle threshold (CT) value of above 35 of the two genes tested by fluorescent quantitative polymerase chain reaction (PCR) is the discharge criteria for mild patients with SARS-CoV-2 in China. Hence, this study was performed to investigate the effect of SARS-CoV-2 vaccines on the time to target CT value (TtCT) greater than 35 for both N gene and ORF gene.

## Methods

### Study Design and Data Source

This is a retrospective cohort study including 142 patients who were locally infected with SARS-CoV-2 Omicron variant on March 2022 in Shanghai, China. They had no or mild symptoms and were admitted to our department for centralized isolation. At admission, the demographic and health information of all patients were collected and saved in the electronic medical records. The SARS-CoV-2 vaccination history of each patient was inquired in detail, including vaccination date, names of vaccine manufacturer, and times of vaccination. This study was conducted in accordance with the Declaration of Helsinki, and its ethical approval was obtained from the Ethics Committee of Shanghai General Hospital, Shanghai Jiao Tong University School of Medicine, Shanghai, China. All participants were informed of the nature and objectives of the study. Informed consent was obtained from each enrolled subject.

### Nucleic Acid Amplification Test of SARS-CoV-2

After nasopharyngeal swab samples were collected, they were shifted to the laboratories immediately and the PCR tests started within 2 h. Real-time fluorescent reverse transcriptase–PCR was used to detect different viral RNA sequences of SARS-CoV-2, including open reading frame (ORF1ab), nucleocapsid (N), and envelope (E) genes. All PCR tests were performed in laboratories of Shanghai Public Health Clinical Center Affiliated to Fudan University, using the SARS-CoV-2 RNA detection kit (Bioperfectus Technologies Co., Ltd., Jiangsu, China) with the SLAN Real-time PCR system following the manufacturer’s instructions. In brief, the total volume of the reaction mixture was 25 μl, and it contained 5 μl of RNA template. The reaction conditions were as follows: reverse transcription at 50°C for 10 min; cDNA pre-denaturation at 97°C for 1 min; denaturation at 97°C for 5 s (45 cycles); then annealing and elongation (with fluorescence monitoring) at 58°C for 30 s; and a final step at 25°C for 10 min. The CT value of each target gene was reported.

The patient received the first nucleic acid amplification test (NAAT) on the second day after admission and the second NAAT on the seventh day after admission. If the CT value was ≥35, then the patient continued to receive the next NAAT in the next day. If the CT value was <35, then the patient received NAAT every other day. Two weeks after admission, NAAT was performed every day.

### Definition and Variables

The latest Diagnosis and Treatment Plan of SARS-CoV-2 (Trial Version 9) was released by the National Health Commission of the People’s Republic of China on March 2022, and the discharge criteria for mild cases were revised as the CT values ≥ 35 of N gene and ORF gene tested by NAAT (fluorescent quantitative PCR with limit value 40) for two consecutive samples with over 24-h interval. In the world, it is generally judged as negative when the CT value exceeds 35. In China, it is judged as negative when the CT value exceeds 40, so as to powerfully reduce the risk of SARS-CoV-2 transmission. The CT value of the new scheme was reduced from 40 to 35, which is limited to the criteria for being out of quarantine and discharge for mild cases after treatment. The adjustment of CT value for discharge is of great significance to improve the allocation of medical resources in China but does not mean that the positive diagnostic criteria for SARS-CoV-2 have changed. TtCT was defined as the duration from the date when the infection of SARS-CoV-2 was first confirmed by NAAT to the date when the patient got two consecutive CT values greater than 35 for both N gene and ORF gene of SARS-CoV-2 with an interval of more than 24 h. Homologous vaccination is defined as multiple injections with the same vaccines and heterogeneous vaccination is defined as multiple injections with different kinds of vaccines.

Hypertension was defined as systolic blood pressure (SBP) ≥140 mmHg, diastolic blood pressure (DBP) ≥90 mmHg, or current antihypertensive therapy. Diabetes mellitus was defined as having a previous diagnosis of diabetes mellitus, receiving oral hypoglycemic agents or insulin treatment, or having a fasting plasma glucose ≥126 mg/dl (7.0 mmol/L) or a hemoglobin A1c level ≥6.5%. The history of coronary heart disease (CHD) was made primarily according to clinical symptoms of angina pectoris, ECG manifestations of myocardial ischemia, and coronary stenosis showed by contrast-enhanced coronary computed tomography angiography or percutaneous coronary angiography. Stroke was defined as a history of cerebral thromboembolism or bleeding manifested by brain computed tomography or magnetic resonance imaging. Renal dysfunction was defined as the estimated glomerular filtration rate (eGFR) <60 ml/min/1.73 m^2^ at baseline or having a history of chronic renal failure.

### Statistics

For numerical variables, the mean (standard deviation) was used for statistical description, and the t-test or ANOVA was performed for inter-group comparison. Categorical data were presented as absolute value and percentage, and they were compared between groups with the chi-square test. All statistical analyses were performed with SPSS 13.0. Two-tailed P-value of less than 0.05 was considered of statistical significance.

## Results

### Demographics and Clinical Data of the Enrolled Subjects

The 142 patients were locally infected with SARS-CoV-2 Omicron variant from March 4 to 30, 2022, in Shanghai, China. Sixty-nine (53.5%) of them were male, and the mean age was 43.1 years (SD, 13.5). The 90.8% of them had been vaccinated before infection. Comparing the vaccinated with the unvaccinated patients, we found no difference in patient characteristics (**Table 1**).

**Table 1:** Patient characteristics.

Variables	TotalN = 142	VaccinatedN = 129	UnvaccinatedN = 13	P*
Age, years old	43.1 ± 13.5	42.9 ± 13.1	46.0 ± 16.9	0.423
Sex male, n (%)	76 (53.6)	69 (53.5)	7 (53.8)	0.980
Hypertension, n (%)	20 (14.1)	18 (14.0)	2 (15.4)	1.000
Coronary heart disease, n (%)	1 (0.7)	0 (0.0)	1 (7.7)	0.092
Heart failure, n (%)	0 (0.0)	0 (0.0)	0 (0.0)	1.000
Stroke, n (%)	0 (0.0)	0 (0.0)	0 (0.0)	1.000
Chronic lung disease, n (%)	0 (0.7)	1 (0.8)	0 (0.0)	1.000
Diabetes, n (%)	4 (2.8)	4 (3.1)	0 (0.0)	1.000
Renal dysfunction, n (%)	0 (0.0)	0 (0.0)	0 (0.0)	1.000
Malignant tumor, n (%)	1 (0.7)	0 (0.0)	1 (7.7)	0.092
Smoking, n (%)	21 (14.8)	19 (14.7)	2 (15.4)	1.000
Body mass index, kg/m^2^	23.8 ± 4.1	23.9 ± 4.1	22.9 ± 4.0	0.394
Times of NAATs	4.2 ± 2.4	4.1 ± 2.3	5.0 ± 3.1	0.186

*P indicated P values when comparing the vaccinated with the unvaccinated. NAAT, nucleic acid amplification test.

### Difference in TtCT Between the Vaccinated and Unvaccinated Individuals

**Figure 1:**
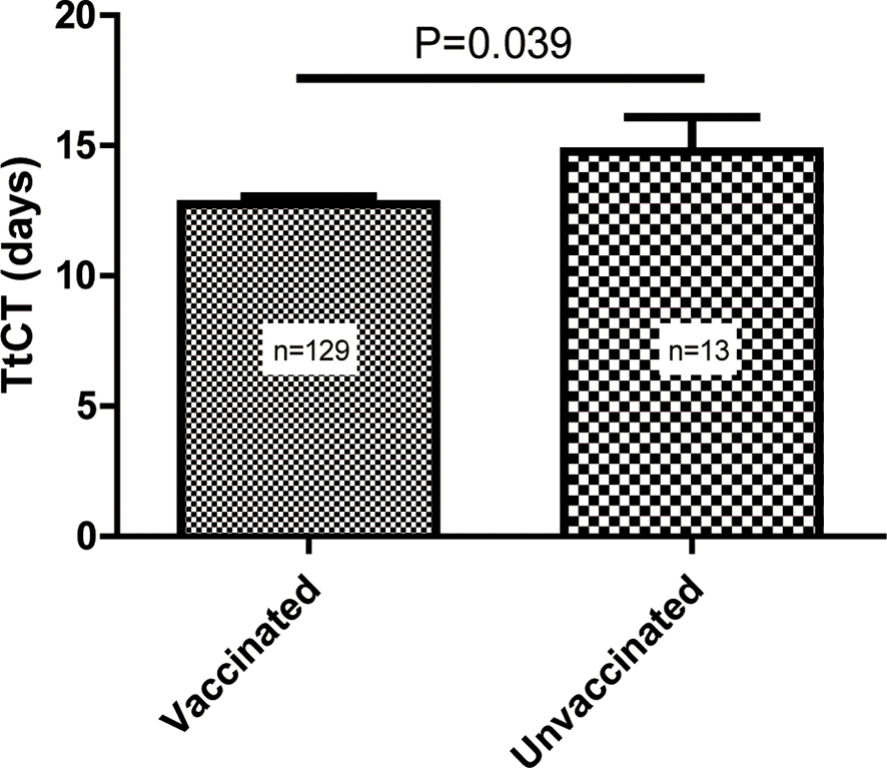
Difference in the time to target cycle threshold value (TtCT) between the vaccinated and unvaccinated individuals.

Compared with patients who did not receive vaccination, patients with vaccination had shorter TtCT (vaccinated vs. unvaccinated, 12.6 ± 3.4 vs. 14.8 ± 4.7 days, P = 0.039) (**Figure 1**).

### Details of SARS-CoV-2 Vaccination for Selected Patients

**Figure 2:**
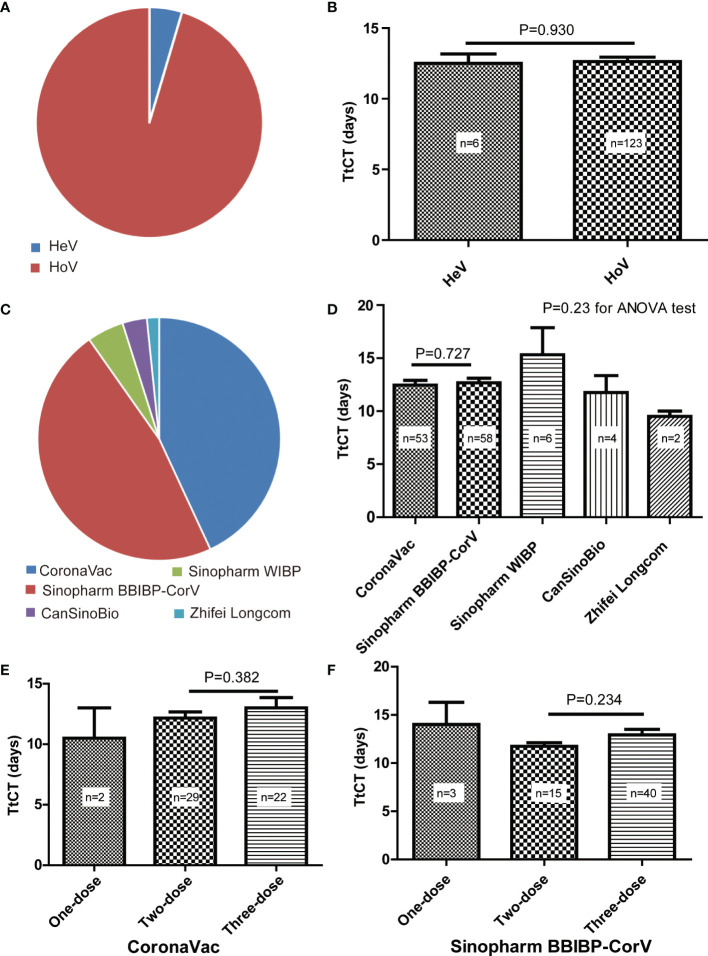
Details of SARS-CoV-2 vaccination for selected patients. **(A)** Proportions of heterogeneous vaccination (HeV) and homologous vaccination (HoV). **(B)** Comparison of the time to target cycle threshold value (TtCT) between HeV and HoV. **(C)** Proportions of different vaccines for homologous vaccination. **(D)** Comparison of TtCT among different vaccines for homologous vaccination. **(E)** Comparison of TtCT between the two-dose primary vaccination and the three-dose booster vaccination of CoronaVac. **(F)** Comparison of TtCT between two-dose primary vaccination and three-dose booster vaccination of Sinopharm BBIBP-CorV.

Overall, we have identified 129 patients with SARS-CoV-2 infection after vaccination, of which 69 (53.5%) of them were male and the mean age was 42.9 years (SD, 13.1) (**Table 1**). Of them, six patients had undergone heterologous vaccination, and the other 123 patients had undergone homologous vaccination (**Figure 2A**). There was no difference in TtCT betweenheterogeneous and homologous vaccination (**Figure 2B**).

For subjects with homologous vaccination, they all were vaccinated with inactivated COVID-19 vaccines produced by the five Chinese manufacturers, such as Sinovac Life Science, Beijing Bio-Institute of Biological Products (BBIBP), Wuhan Institute of Biological Products (WIBP), CanSinoBio, and Zhifei Longcom. Patients vaccinated with CoronaVac (Sinovac Life Science) accounted for 43.1%, Sinopharm BBIBP-CorV for 47.2%, Sinopharm WIBP for 4.9%, CanSinoBio for 3.3%, and Zhifei Longcom for 1.6% (**Figure 2C**). No difference in TtCT was observed among different vaccines (**Figure 2D**).

When comparing two-dose primary vaccination with three-dose booster vaccination, we found no difference in TtCT for both CoronaVac (**Figure 2E**) and Sinopharm BBIBP-CorV (**Figure 2F**) respectively.

## Discussion

The main findings of this study are as follows: 1) Vaccination is associated with shorter TtCT in patients with SARS-CoV-2 Omicron variant; 2) inactivated vaccines produced by different manufacturers in China have similar effects on TtCT; and 3) compared with the two-dose primary vaccination, the three-dose booster vaccination does not appear to reduce TtCT.

Currently, vaccination has been verified to be the most effective way to prevent SARS-CoV-2, irrespective of the mRNA or the inactivated vaccines (Fan et al., 2021). Although the SARS-CoV-2 vaccines currently used could not powerfully prevent infection, it significantly reduced the incidence of severe cases after infection. Two inactivated SARS-CoV-2 vaccines, CoronaVac and BBIBP-CorV, were developed in China and were reported to reduce the risk of symptomatic SARS-CoV-2 with rare serious adverse events (Al Kaabi et al., 2021; Tanriover et al., 2021). Although these two vaccines have been widely used in China, their phase 3 trials were not performed in China, for there were few individuals infected with SARS-CoV-2 after the second half of 2020 in China due to the Chinese dynamic clearance strategy and strict adherence to public health measures. It was impossible to complete the enrollment at that moment in China. Thus, actually, less is known about their effectiveness in the Chinese population. In addition, there is a lack of knowledge about their roles in protection against SARS-CoV-2 Omicron variant in China. This study shows that the vaccinated patients had shorter TtCT than the unvaccinated ones, indicating the inactivated vaccines keep working effectively against SARS-CoV-2 Omicron variant in the Chinese population. The mutations are causing numbers of SARS-CoV-2 variants that confer escape from antibodies (Kontopodis et al., 2022). There is no guarantee that the disease caused by subsequent mutations will be less severe and that the previous vaccines are still effective. Hence, it is of great significance to pay continuous efforts to evaluate the effectiveness of the previous vaccines against the new SARS-CoV-2 variants. As the SARS-CoV-2 continues to evolve, humans must be ready to develop new vaccines at any time.

A meta-analysis of different vaccines at phase 3 indicated that mRNA vaccines conferred a lesser risk of SARS-COV-2 infection and showed more relevance to serious adverse events than viral vector and inactivated vaccines (Fan et al., 2021). A higher humoral response rate was also observed after using the mRNA vaccine compared with the inactivated vaccine (Lu et al., 2022; Ciampi et al., 2022). This suggests that there are great differences in the safety and effectiveness of different kinds of vaccines. For vaccines of the same kind but developed by different manufacturers, it is unclear whether such difference also exits. This retrospective cohort showed that most individuals suffered from this wave of SARS-CoV-2 pandemic in Shanghai, China, had been vaccinated with CoronaVac (Sinovac Life Science) accounting for 43.1% and Sinopharm BBIBP-CorV accounting for 47.2%. Our data indicated that there was no difference in TtCT between these two inactivated vaccines. This may suggest that the homogeneity of these two vaccines is good in the Chinese population.

The inactivated SARS-CoV-2 vaccines have been widely used in a two-dose schedule (Tanriover et al., 2021; Tanriover et al., 2021). A third vaccine booster dose appears to induce a significant increase in binding and neutralizing antibodies, which may improve protection against infection (Costa Clemens et al., 2022). However, our results show that the booster vaccination does not appear to reduce TtCT compared with the two-dose primary vaccination. Therefore, we hypothesize that the booster vaccination may increase antibody titer in a short time, but such effect would gradually decline. It is also unknown whether it is necessary to take the fourth or fifth dose. Hence, a randomized controlled trial is expected to evaluate whether the three-dose booster vaccination is superior to the two-dose primary vaccination.

In conclusion, vaccination is associated with shorter TtCT in patients with SARS-CoV-2 Omicron variant. It indicates that the inactivated vaccines keep working effectively against SARS-CoV-2 Omicron variant in Chinese population.

## Data Availability Statement

The raw data supporting the conclusions of this article will be made available by the authors, without undue reservation.

## Ethics Statement

The studies involving human participants were reviewed and approved by Ethics Committee of Shanghai General Hospital, Shanghai Jiao Tong University School of Medicine, Shanghai, China. The patients/participants provided their written informed consent to participate in this study.

## Author Contributions

Conceptualization: YW; methodology: YW and JW; formal analysis: YW; investigation: YW, JW, FS, SZ, YL, XT, and PZ; writing: YW. All authors contributed to the article and approved the submitted version.

## Conflict of Interest

The authors declare that the research was conducted in the absence of any commercial or financial relationships that could be construed as a potential conflict of interest.

## Publisher’s Note

All claims expressed in this article are solely those of the authors and do not necessarily represent those of their affiliated organizations, or those of the publisher, the editors and the reviewers. Any product that may be evaluated in this article, or claim that may be made by its manufacturer, is not guaranteed or endorsed by the publisher.
